# Microspectrometric insights on the uptake of antibiotics at the single bacterial cell level

**DOI:** 10.1038/srep17968

**Published:** 2015-12-11

**Authors:** Bertrand Cinquin, Laure Maigre, Elizabeth Pinet, Jacqueline Chevalier, Robert A. Stavenger, Scott Mills, Matthieu Réfrégiers, Jean-Marie Pagès

**Affiliations:** 1 DISCO beamline, Synchrotron Soleil, Saint-Aubin, France; 2 UMR-MD1, Aix-Marseille Université, IRBA, Marseille, France; 3 Antibacterial DPU, GlaxoSmithKline, Collegeville PA, USA; 4 Infection Bioscience, AstraZeneca R&D Boston, Waltham, Massachusetts, USA

## Abstract

Bacterial multidrug resistance is a significant health issue. A key challenge, particularly in Gram-negative antibacterial research, is to better understand membrane permeation of antibiotics in clinically relevant bacterial pathogens. Passing through the membrane barrier to reach the required concentration inside the bacterium is a pivotal step for most antibacterials. Spectrometric methodology has been developed to detect drugs inside bacteria and recent studies have focused on bacterial cell imaging. Ultimately, we seek to use this method to identify pharmacophoric groups which improve penetration, and therefore accumulation, of small-molecule antibiotics inside bacteria. We developed a method to quantify the time scale of antibiotic accumulation in living bacterial cells. Tunable ultraviolet excitation provided by DISCO beamline (synchrotron Soleil) combined with microscopy allows spectroscopic analysis of the antibiotic signal in individual bacterial cells. Robust controls and measurement of the crosstalk between fluorescence channels can provide real time quantification of drug. This technique represents a new method to assay drug translocation inside the cell and therefore incorporate rational drug design to impact antibiotic uptake.

Multi-drug resistant (MDR) Gram-negative bacteria such as *Escherichia coli* (*E. coli*) and other *Enterobacteriaceae* are spreading rapidly and in many cases are capable of producing severe infections that can eventually lead to death (135 000 infections in Europe and 215 000 in the USA annually) and contributing to the concept of the ESKAPE alert in clinical bacteriology and emerging pathogens^1^,^2^. The widespread use, and misuse, of antibiotics results in the generation/release of antibiotic concentration gradients in the environment, including humans, animals, water, etc^3^,^4^. Consequently, bacteria are frequently exposed to subinhibitory concentrations of antibiotics leading to the evolution, selection and potential spreading of antibiotic resistance^3^,^5^,^6^. Different resistance mechanisms have been highlighted and include: (i) drug inactivation or modification by enzymatic action (*e.g.* ß-lactamase, acetylase). (ii) Alteration/mutation or masking of targets (*e.g.* penicillin binding proteins, Type II topoisomerases). (iii) Alteration of metabolic pathways: bacteria resist antimicrobial agents by using alternate pathways than those inhibited by the molecule, or bacteria increase the production of the target metabolite (*e.g.* using pre-synthesized folic acid, increasing the rate of folic acid synthesis). (iv) Changing the membrane permeability (*e.g.* downregulation of porins, overexpression of efflux pumps). Employing this last strategy, the bacterium is able to manage the intracellular concentration of antibiotics by modulating the entry or the ejection of active agents^7^. Thereby, the effective concentration of drug is never reached inside the cell and consequently its activity is minimized. Furthermore, the relatively low concentration of antibiotic inside the bacterium can promote adaptation by developing the expression/selection of other resistance mechanisms^8^,^9^. Thus, the “multi” in the term MDR can be read at different levels: multi because of the multiple antibiotic classes a bacterium can be resistant to, but also because multiple and various mechanisms contribute to the bacterial survival in the presence of antibacterial agents^10^. Deciphering the biochemical basis and the mechanistic processes underlying the accumulation of antibacterial agents is essential to design and develop antibiotics that can achieve higher, more effective intracellular concentrations and avoid further spreading of resistance. This is particularly important with the continuing emergence and the worldwide distribution of MDR bacteria and the paucity of new antibacterial agents to treat MDR bacteria^11^,^12^,^13^. The paper of Laxminarayan *et al.*^14^ and the resulting comments clearly illustrate the acute intensity and therapeutic relevance of this point.

A key unmet point is the ability to measure the quantity of antibiotic inside the bacterium. Several experimental technologies have been developed to reach this goal, for example: bioassays, fluorimetric detection, radiometry, and HPLC. These techniques, although having important benefits, fail to give a resolution on the mechanisms involved in drug penetration or efflux since they are measuring a population of bacteria and often require chemical modification of the antibiotic to render it detectable, e.g. a fluorescent tag, radioelements. Ultraviolet absorption is label-free but lacks appropriate sensitivity, concentrations in the microgram per milliliter range are necessary for a strong signal. Chapman and Georgopapadakou^15^ proposed UV fluorimetry. Based on the natural fluorescence by UV excitation of antibiotics such as ciprofloxacin and compound **1**, an original approach has been developed by using deep UV (DUV) to study the drug accumulation inside MDR bacterial suspensions^16^. But, DUV excitation of bacteria leads also to a convoluted signal due to emission of bacterial fluorophores such as tryptophan, tyrosine or NAD, together usually referred to as autofluorescence, which can interfere with the signal from the antibiotic. This label-free approach is sensitive and can be performed not only by spectrofluorimeters but also by conventional microscopes. Studies using spectrofluorimetry carried out on a bacteria population (a bacterial cell suspension) or on lysates can provide rapid results, but fail to describe the individual concentration and antibiotic localization.

We report here an innovative method for quantifying the kinetics of antibiotic accumulation at the single bacterium level and the role of membrane-associated mechanisms of resistance on this process. The techniques previously described^17^,^18^ combine the spatial resolution of microscopy with the high spectral definition required to distinguish drug-related signal from autofluorescence due to the UV excitation. Deep ultraviolet (DUV) photons, provided by synchrotron radiation, bring excellent tuneability and sufficient flux to follow label-free antibiotics.

## Results

**Figure 1:**
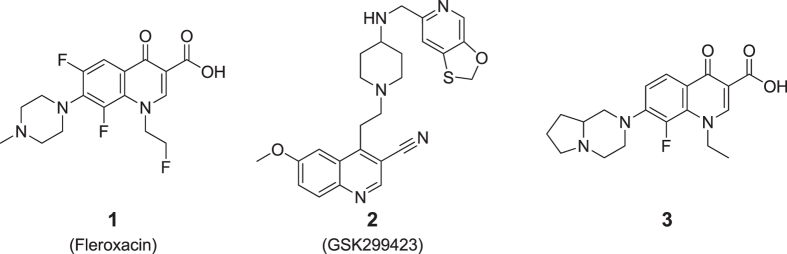
Chemical structures of the antibacterial agents used.

In the present study, compounds **1–3** were selected to demonstrate the potential of this method. Fleroxacin (compound **1**) is a well characterized fluoroquinolone antibiotic while compounds **2**^19^ and **3** represent an entirely novel class of topoisomerase inhibitor and a novel quinolone topoisomerase inhibitor respectively (Fig. 1 and Supplementary Figure 1). These were selected for their spectral compatibility with our protocol in addition to an appropriate biological assessment of their activity on selected MDR *Enterobacter aerogenes* clinical isolates. Indeed, the determination of their Minimal Inhibitory Concentration (MIC) showed, as expected, that their whole cell antibacterial activity can be modulated by addition of a membrane permeabilizer, such as polymyxin B nonapeptide (PMBN), an efflux blocker such as phenylalanine-arginine-β-naphthylamide (PAβN) or an energy poison, carbonyl cyanide *m*-chlorophenylhydrazone (CCCP). These three adjuvants presumably modulate the influx or efflux respectively of compounds **1–3** (Table 1). *E. aerogenes* clinical isolate EA289, which has been shown to overexpress AcrAB efflux pumps, and its efflux deficient *tol*C^-^ derivative EA298, have previously been used as model systems for MDR bacteria^16^,^20^,^21^. Importantly, these two strains contain mutations in the quinolone target (QRDR region of DNA gyrase) that strongly contribute to the quinolone resistance and consequently, even when we completely blocked the efflux mechanism by adding PAßN or CCCP, we cannot totally restore the susceptibility for antibiotics that target DNA gyrase^16^,^20^.

To assess the role of efflux in intrabacterial accumulation, incubation was also carried out in the absence or in the presence of CCCP that collapses the energy-driven force needed by efflux pumps^21^,^22^.

### Need and Strategy for Segmentation and Sorting

**Figure 2:**
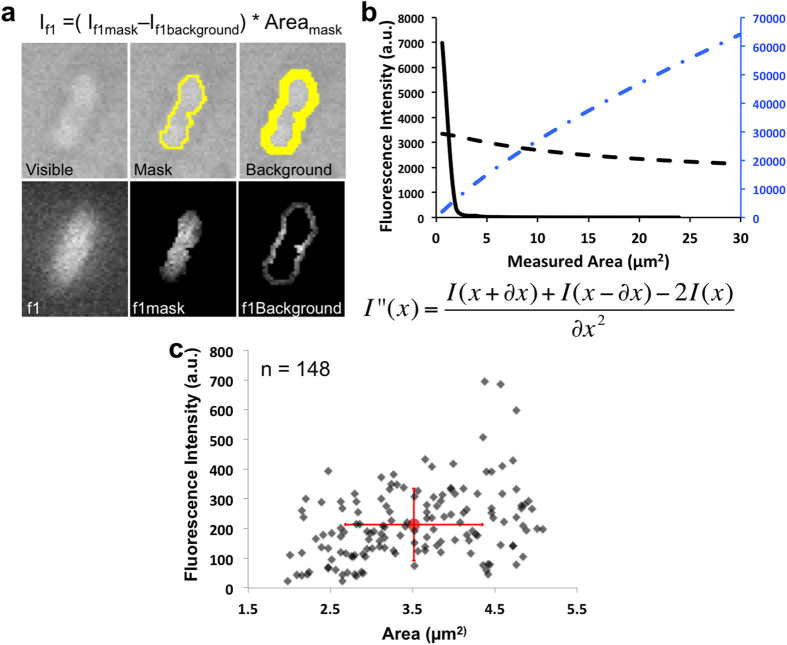
Strategy for Segmentation and Sorting. (**a**) Images acquired on DUV microscope. Presented on top panels from left to right, a bacterium under « visible » transmitted light, the automatic computed mask and the background mask. Shown on lower panels, the emission intensity of bacterium under 275 nm excitation (30 sec acquisition) in the filter 1 (327–353 nm) that select the autofluorescence. To measure the fluorescence of the bacterium, its total intensity of fluorescence is subtracted by the average intensity selected by the background mask reported for the whole bacteria and divided by the bandwidth of the used filter. (**b**) Total Intensity of fluorescence depending of area centered on a bacterium (blue dotted line), Average intensity of fluorescence (black dotted curve), Second derivative of the total intensity of fluorescence calculated using the formula written under the graph (black curve). (**c**) Fluorescence intensity from autofluorescence channel for each bacterium against size.

In order to obtain reliable measurements on each individual bacterium, segmentation of the signal needed to be carried out to provide an appropriate ‘outline’ of the cells. In the present case, the bacterium covered a relatively small area; therefore the amount of information per pixel was sensitive to the area selected for analysis. Even using a 100 X objective, the bacterium was only represented by a few hundred pixels. Manually drawing a contour around the cell led to bias that under- or over-estimated the area of the bacterium (See remark on Fig. 2).

Therefore, the key point of this method was to segment each bacterial cell in a consistent, reproducible and relevant manner. To address this problem, we augmented the DUV-fluorimetry data acquisition with two other transmitted light images, taken before and after the DUV-fluorimetry acquisition (Fig. 2a). The first transmitted light image generated well-defined borders bacterial cells with, which provided a boundary to segment the cells. The second transmitted light image was used to focus the analysis on only the immobile bacteria. Bacteria masks were automatically created using a background correction and a threshold on the visible images then reported on both fluorescence images. Centers and contours for each bacterium were then computed (Fig. 2a). Given the heterogeneity of illumination in this system, applying a simple threshold was not appropriate. The image was first homogenized using Enhanced Local Contrast (CLAHE^23^). Next the background was subtracted with a “rolling ball”^24^. Finally, a simple smoothing was performed to obtain better segmentation using maximum entropy thresholding^25^. To discard any other fluorescent elements that did not have the appropriate rod shape, and therefore were not originating from a bacterium, we used the plug-in IJBlob^26^,^27^. This allowed the selection of elements with an elongation defined by the difference from the ratio between the smaller side and the larger side of a square (ratio is 1) and the shape defined by the segmentation. Other features such as the Feret diameter allowed straightforward measurement of the bacterium length and its morphological plasticity. We then measured the variation in fluorescence intensity of the bacterium center’s isomorphic region (Fig. 2b). The second derivative of the blue dotted curve showed an inflexion point (Fig. 2b, black curve) around 2.5 squared microns, which is consistent with the size of a bacterium. We used the latter to check the quality of our segmentation approach. Poor segmentation led to incoherent cell surfaces. Secondary masks were generated around each bacterium (presented in Fig. 2a) in order to correct for heterogeneity in the excitation beam by selecting a local background. The average intensity of the secondary mask was used to correct the background for each bacterium (formula under Fig. 2a). The total intensity obtained with each bacterium was automatically calculated and recorded. Intensity was then normalized by the bandwidth from the filter after background correction. Following this, another sorting procedure was applied to remove any bacteria with negative intensity. Also, when two bacteria were too close to each other during the measurement, the background for one bacterium was tainted by the fluorescence of the second, hence both bacteria were discarded. Measurements were performed only in bacteria with an area between 70 and 300 pixels to avoid adding noise from fragments, unfocused, or aggregated bacteria.

### Comparison of intensities from the two channels: Crosstalk

**Figure 3:**
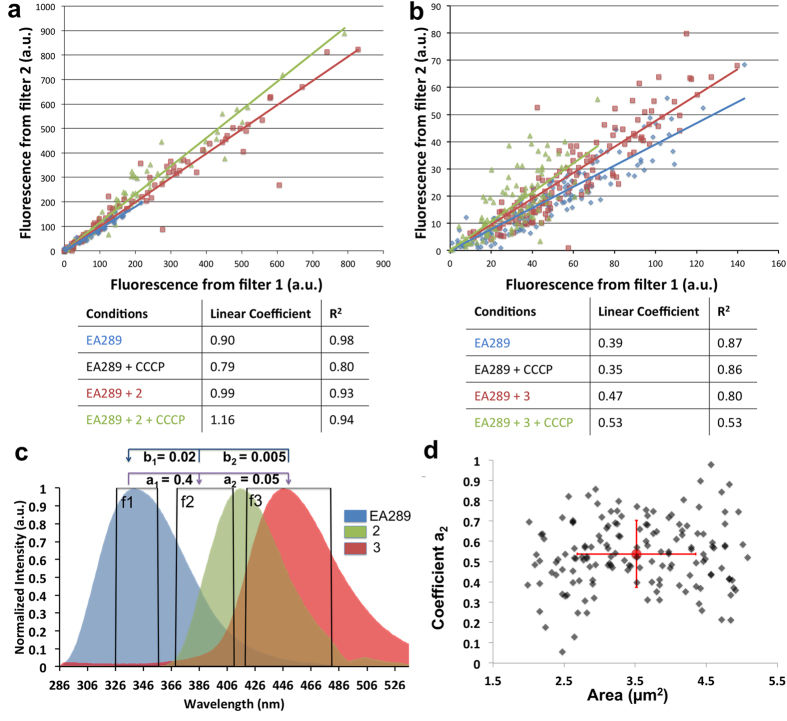
Analyses of intensities from the two channels. (**a**,**b**) Graphs represent the fluorescence measured by filter 2 in function of fluorescence measured by filter 1. Three conditions are represented for each compound (**a**) for compound **2** and (**b**) for compound **3**: EA289, EA289 with compound and EA289 with compound and CCCP. The condition EA289 with CCCP has not been represented on the graph for a better clarity. The table presents the fitting parameters of the populations by a line, the linear coefficient and the coefficient of determination. (**c**) Emission Fluorescence Spectrum of Bacteria, compounds **2** and **3** with excitation wavelength of 275 nm. The spectra have been normalized by the maximum. f1, f2 and f3 squares symbolize the used filter for autofluorescence (327–353 nm), **2** (370–410 nm) and **3** (420–480 nm). Crosstalk coefficient of f1 in f2 and f3 are respectively called a_1_ and a_2_. Crosstalk coefficient of f2 in f1 and f3 in f1 are respectively called b_1_ and b_2_. We remark that b coefficients are significantly smaller than a coefficients. (**d**) represents the heterogeneity of the parameter a for compound **2** depending of the bacterium size (black dots with 30% transparency).

Relating autofluorescence from the channel defined by filter 1 and drug fluorescence recorded in the channel called filter 2, may give information on drug accumulation. The intensity of both channels for each bacterium were normalized by subtracting background values and dividing by the spectral bandwidth of the respective channel. Intensity measured in the channel 2 was compared to the intensity measured in the channel 1 for each bacterium. We obtained the population presented in Fig. 3. For the control assay (bacteria alone, no antibiotic), we assumed that the intensity measured in the channel 2 was proportional to the intensity measured in the channel 1 since it was due to leakage of fluorescence from one filter to another, otherwise known as crosstalk. Taking this characteristic into account, the data were fitted linearly.

Two additional parameters were important to consider: the director coefficient and the coefficient of determination. The first is an indicator of the crosstalk coefficient; the second is a measure of the fitting quality. We expected to find a stable ratio and a good linear fit across the untreated bacteria (Fig. 3). The crosstalk coefficient was indeed independent of the autofluorescence intensity. We assumed that in experiments with added drug, the intensity measured in the channel 2 due to drug accumulation was not related (i.e. not proportional) to the intensity measured in the channel 1. As a result, we measured a higher director coefficient and a lower coefficient of determination since no prediction of fluorescence emitted in the channel 2 could be performed using only the measure of the channel 1.

When the bacteria were incubated with compound **3** (Fig. 3b), we observed a linear relationship for the two fluorescence quantities and a higher linear coefficient than that obtained in the absence of the compound. In the presence of CCCP, this coefficient was even higher. This indicated that the signal due to compound **3** entering the bacterium was proportional to the level of autofluorescence, with or without CCCP. As shown in the Figure S2, CCCP had no fluorescence under the experimental conditions and did not alter the fluorescence of the studied compound. For compound **2**, we observed the presupposed behavior, a higher linear coefficient and a lower coefficient of determination. However, we observed a significant effect of CCCP on the accumulation of compound 2 (Fig. 3a). The amount of the signal obtained with compound **2** in the bacteria was less dependent on the level of autofluorescence which suggested different activity inside cells.

### Crosstalk Correction

The crosstalk effect is a well known phenomenon first recognized and corrected in cytometric studies^28^ and later in FRET measurements. For conventional studies, crosstalk is rarely an issue since the emission wavelengths are spectrally distinct. In addition, mathematical treatment often raises the noise without achieving better signal separation. In our case, correcting for crosstalk was fundamental since the emissions were close to each other in space with significant overlap between the autofluorescence emission and the antibiotic emission as presented in Fig. 3c (emission spectrum). Measuring the autofluorescence heterogeneity for each bacterium in a given sample population was important in order to differentiate the link between drug accumulation and autofluorescence (Fig. 2).

**Figure 4:**
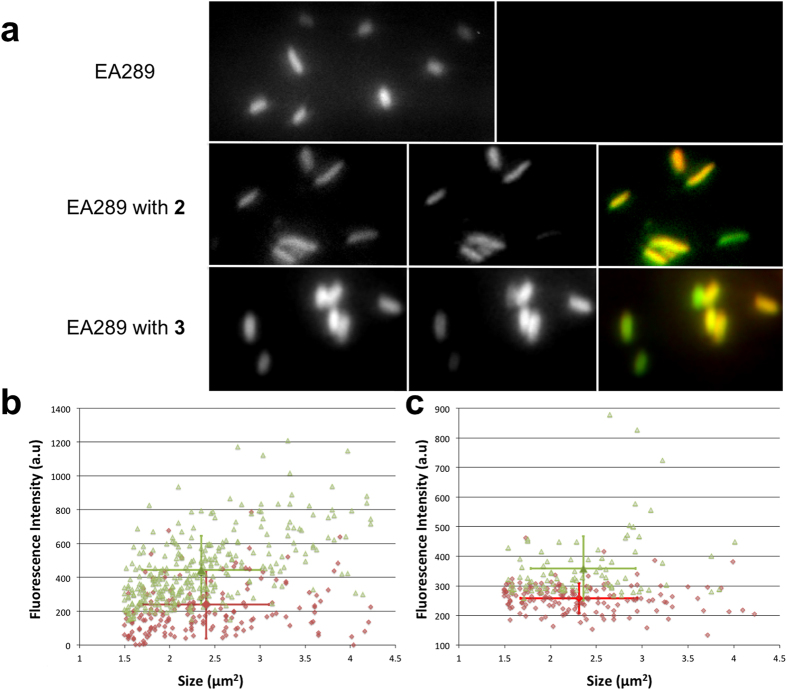
Crosstalk Correction and final fluorescence intensity. (**a**) Fluorescence images of EA289 bacteria excited with a wavelength of 275 nm, untreated (top panel), incubated with compound **2** (middle panel) or with compound **3** (lower panel): emission from filter 1 (327–353 nm; left panel); emission from filter 2 (370–410 nm for compound **2** and 420–480 nm for compound **3**; middle panel) corrected from crosstalk effect; merge of the two precedent images : filter 1 in green, filter 2 in red (right panel). (**b**) Fluorescence corrected from crosstalk effect in function of its size from each bacterium treated with compound **2** (red diamonds) or with compound **2** + CCCP (green triangles) and (**c**) with compound **3** (red diamonds) or with compound **3** + CCCP (green triangles). The red dots with bars and green dots with bars are the average with standard deviations for each population.

In order to measure the emission of the antibiotic alone, we first performed image acquisitions of untreated bacteria to measure the extent of autofluorescence collected by filter 2 (Fig. 4a). Secondly, we acquired a second set of images with bacteria incubated with drugs at different time points in the presence or absence of the efflux blocker CCCP.

We defined the crosstalk coefficient from the channel 1 in the channel 2 as **a,** and the coefficient from the channel 2 in the channel 1 as **b** and intensity of a given signal *s* was ‘**I**_**s**_”. Considering (i) that filter 1 primarily collected the autofluorescence signal and limited amounts of the fluorescence signal from the antibiotics, and (ii) that filter 2 primarily collected the fluorescence signal from the antibiotic and limited amounts of the autofluorescence, we formulated the following equation:


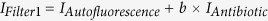
 and 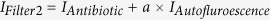 where b and a corresponded to the crosstalk coefficients between the filters used to measure the autofluorescence and the filters used to measure the fluorescence emitted by the compound.

We considered the following sets of equation with another pair of parameters α and β: 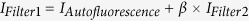 and 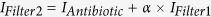 But this is not correct. This treatment denies the fact that the intensity in each filter is respectively a composition of intensity from the autofluorescence and the drug fluorescence.

Which leads to 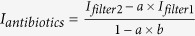 These coefficients were first evaluated from the normalized recorded spectra since it was impossible to know *a priori* the coefficient for a tested bacterium that had accumulated different amount of antibiotics. Because the heterogeneity of bacterial autofluorescence was significantly different of the fluorescence emitted by the compound, the ‘b’ coefficient was always about 10 to 20 times lower than the ‘a’ coefficient. The product a x b was insignificant and had no impact on the correction (Fig. 3c).


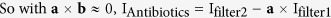


Because our studies focused on the individual bacterium rather than a population and that a fluorescence microscope had a significantly different optical path relative to a spectrofluorimeter, **a** needed to be properly measured on the DUV microscope before image analysis for each experiment (Fig. 4).

Coefficients measured using the microscope were indeed different from the spectrofluorimeter with a_2_ ~ 0.90 for compound **2** and a_3_ ~ 0.35 for compound **3** (Fig. 3c). The parameter **a** presented a heterogeneity as shown in Fig. 3d, Therefore, it was not possible to apply a perfect parameter for each individual bacterium. Rather, an average coefficient was calculated and applied to correct the crosstalk effect.

This average crosstalk value impacted the heterogeneity of the measure. However, as we showed that the average crosstalk is not bacterium size-dependant (Fig. 2), we do believe that we minimized the impact of crosstalk corrections. Using the minimum crosstalk correction value increased the number of bacteria that accumulated antibiotics and raised the standard deviation for the antibiotics fluorescence by about 10%. On the other hand, using the maximum correction value decreased the number of bacteria with a positive value (more bacteria with negative fluorescence would be measured and so these were discarded). The heterogeneity would therefore be decreased by about 10%.

Figure 4a presents the correction carried out on all acquired images. After incubation for 30 min with either compound **2** or **3**, heterogeneous intensities observed were not proportional to the autofluorescence level. Analysis of each bacterium in the absence or in the presence of the efflux inhibitor CCCP was also performed. The populations are represented in Fig. 4b,c. For each compound tested, the fluorescence obtained was not proportional to the bacterial size, suggesting that compound accumulation was different for each bacterium. When the average fluorescence value was considered, we observed a clear increase of drug accumulation in the presence of CCCP for both compounds. This effect was confirmed by e spectrofluorimetric studies of lysed bacteria after 30 min of incubation under the same conditions (Table 2).

### Correction of Photobleaching and Photoadducts

**Figure 5:**
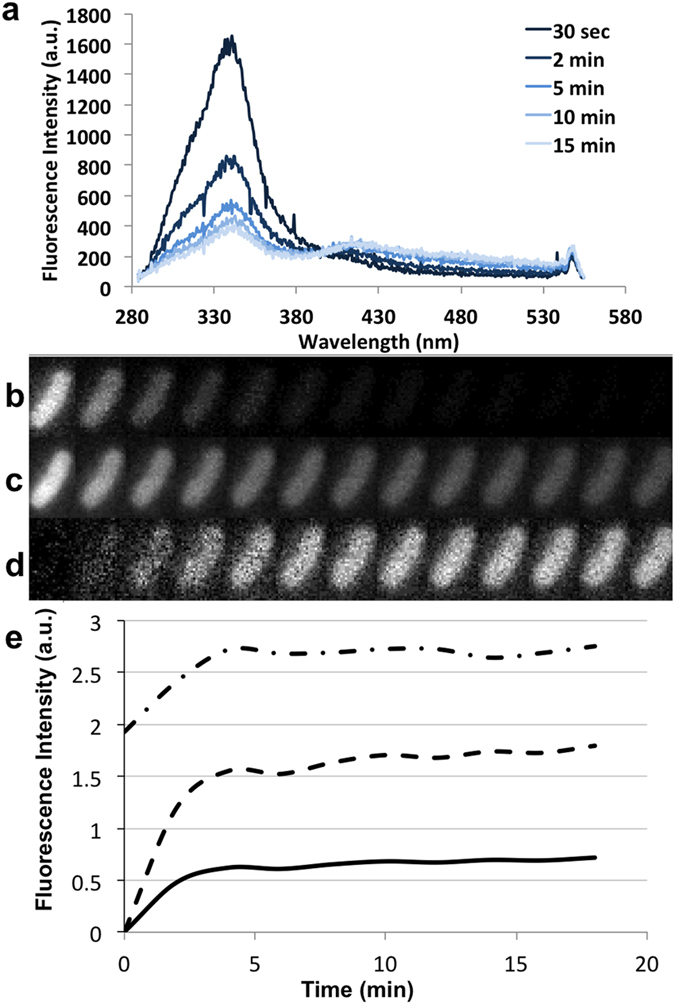
Correction of Photobleaching and Photoadducts. (**a**) Average Spectrum of 5 single bacterium under 275 nm excitation acquired every 30 seconds. Only the spectrum 30 sec, 2 min, 5 min, 10 min and 15 min have been presented. The time dependant photobleaching, and photocreations may be seen. (**b**) Fluorescence intensity by bacterium accumulating compound **2** (one frame every two minutes) emitted between 327 and 353 nm. **c** Fluorescence intensity by bacterium accumulating compound **2** (one frame every two minutes) emitted between 370 and 410 nm. (**d**) is (**c**) with background, photobleaching and crosstalk corrections. (**e**) Fluorescence intensity of the bacterium from background corrected images (dashed and dotted line, thumbnails (**c**) from background and photobleaching correction (dashed lines) and from background, photobleaching and crosstalking corrections (solid line, thumbnails (**d)**).

UV excitation data rarely provides a straightforward fit for biological experiments given the complexities of the systems involved, and the current studies were no exception. Therefore, although single point measurements were possible without further correction, measuring and controlling the effects of the UV radiation was necessary during time course experiments. *E. aerogenes* EA289, which overproduces the AcrAB-TolC efflux pump, was plated and spectra of single bacteria were acquired using the DUV microspectrofluorimeter. Consecutive snapshots at 30 sec intervals were recorded, as shown in Fig. 5a. Although photobleaching of the tryptophan peak was expected, increased of fluorescence detected between 400 and 500 nm was not. Complementary experiments were been carried out to understand the origin and nature of this fluorescence.

Generation of reactive oxygen species by UVB irradiation has been heavily studied^29^,^30^. Tryptophan is the most relevant contributor for autofluorescence and also the most photoreactive. UV irradiation has been shown to be responsible for the photochemical cleavage of tryptophan into *N*-formylkynurenine (NFK)^31^,^32^. NFK is also excited with UVBs with peak emission at approx. 440 nm. In reality, more than one photoproduct is generated and the observed emission is a blend of NFK species^33^.

Knowing this, we performed time-lapse experiments on the DUV microscope. Bacteria were plated and observed for 20 min with a sampling time of 2 min: 30 sec with the filter 1, 30 sec with the filter 2, followed by 60 sec of pause. Thumbnails of a bacterium incubated with compound **2** are shown Fig. 5b,c. During the intervening pause in sampling, the same acquisition cycle was performed on another bacterium, avoiding constant UV irradiation of the same field. The DUV microscope provides a wide-field excitation while the DUV microspectrofluorimeter provides a confocal effect. Thus, the total amount of UV irradiation is different. Photobleaching in the channel 1 corresponding to autofluorescence was observed (Fig. 5b). In the channel 2 (Fig. 5c), bleaching was also observed but was not proportional to the measured intensity from the channel 1 for the different acquired images. For filter 2, the signal increased over time and was not correlated to photobleaching. For dynamic studies, control experiments were needed to generate appropriate time-course correction values for normalization. Photobleaching correction was not done independently of the occurring photocreation. Therefore, the average crosstalk coefficient measured along time was systematically used during the measure for correction. The previously described method was used to analyze the time dependent crosstalk coefficient with an assumption that at time 0, no compound had accumulated. Therefore, the intensity in channel 2 for compounds **1**–**3** was the same as the control experiment and provided an origin for accumulation. By reporting this parameter in the drug accumulation experiments, we were able to correct for both the photobleaching and the crosstalk (Fig. 5d,e).

Since the bacteria were incubated with drugs, the background fluorescence might have evolved during the accumulation time-course. Concerns have been raised regarding the fluorescence spectra of the compound outside versus inside the bacteria. Previous spectromicroscopy studies^16^ have indicated that the bacterial environment does not change the emission spectrum. Further, the measured background intensity remains the same in the absence or in the presence of tested drugs due to our threshold of detection, as drugs have to reach a certain concentration to be observed in a single bacterium.

### Time-course accumulation in individual bacteria

We carried out similar analyses as above in order to better understand the impact of antibiotic influx and efflux on the accumulation of compounds **1**–**3**. Compound **1** was used as a reference compound because we had accumulation data from our previous work^16^. Immediately after addition of the antibiotic to the cells in buffer, 0.5 μL of the mix was introduced between two quartz coverslips. For better comparison between the experiments, the first measurement was taken three minutes after addition of antibiotic to allow time to adjust the initial focus.

**Figure 6:**
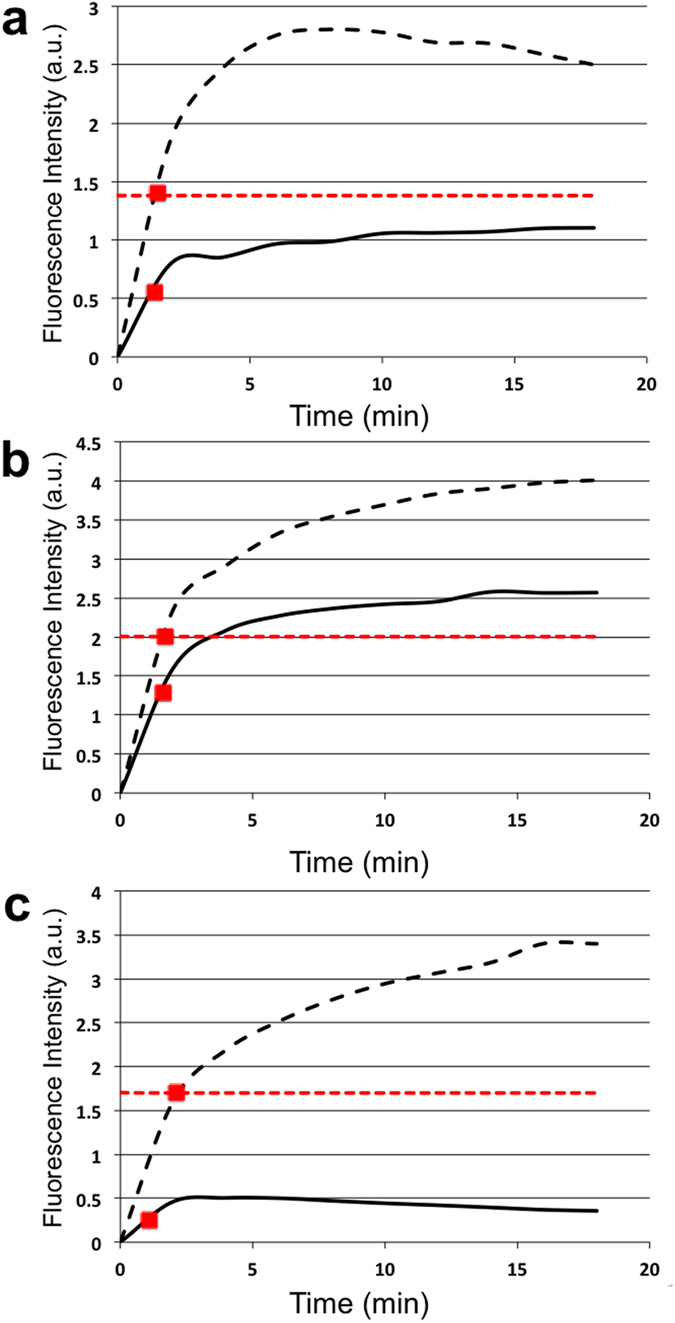
Time course of bacteria accumulation. Average Fluorescence intensity of 100 individual EA289 (solid line) or EA298 (dashed line) bacteria incubated with compound **1** (**a**), **2** (**b**) and **3** (**c**). We consider that the maximum (steady state) of accumulation is reached at 20 min in EA298. 50% of accumulation is presented as the horizontal dashed red line. The red dots presents 50% of accumulation when the plateau is reached for each strain.

In order to determine how changes in efflux affected drug accumulation, strains that either overproduced the AcrAB-TolC pump or did not produce TolC (TolC null mutant) were used. Both strains being isogenic, we hypothesized that the difference of the accumulation kinetics would be due only to the efflux transporter activity. Figure 6 presents the time course of accumulation of compounds **1**, **2** and **3** in the efflux-overproducer (EA289) and efflux-deficient (EA298) strains. In EA289 a lower signal, corresponding to less accumulation, was observed for each compound when compared with the data for EA298. Further, with EA298 each compound accumulated at different rates and reached different steady-state levels. With compound **1** (Fig. 6a), a plateau was reached after 15 min. With compound **2** (Fig. 6b), a plateau was reached around 8 min in EA298 vs. EA289. With compound **3** (Fig. 6c), a plateau was reached at long incubation time (>15 min). When the incubations were carried out in the presence of CCCP, the accumulation of compound **3** reached the same plateau in EA289 and EA298 at a similar level observed for EA298 without CCCP (Supplementary Figure S3). For compound **3**, the accumulation was faster in both strains than without CCCP and reached the level observed in the efflux knockout strain EA298. For these molecules, the accumulation rate in EA289 was strongly increased in the presence of CCCP reflecting the involvement of efflux pumps in reducing the intrabacterial antibiotic accumulation.

To test the robustness of this method, results obtained with the time course experiments were compared to those obtained with the bacterial population treated under similar conditions and lysed after 30 min of incubation (Table 2). The kinetic curves and the respective accumulation level were in agreement with the lysate experiment. With compound **3**, the results obtained by the two methods were similar in EA289 + CCCP and EA298 +/− CCCP, and a 2-fold increase of the accumulation was observed in EA289 in presence of CCCP (Supplementary Table S1).

## Discussion

To gain a better understanding of the dynamics of drug accumulation, we have developed a method to follow the antibiotic concentration over time in multiple individual bacteria. This approach on single bacterial cells has not been reported and represents a significant technical advance in the field.

A challenge is that we cannot irradiate the bacteria for an extended time period which limited the sampling times during the accumulation experiments. The first consequence is a relatively low signal to noise ratio. For this reason, correcting for crosstalk was necessary and therefore it was important to measure and quantify the amount of fluorescence provided by the drug itself and not by the bacteria. Our method for correction was based on generating masks from transmitted light images that allowed us to distinguish between the mobile and immobile bacteria (Fig. 2). Correlation of the drug accumulation and the bacterial shape or the bacterial division state by developing morphological tools will be considered in the next steps of this work.

Addition of CCCP during the experiments allowed us to investigate the role that efflux pumps play in compound accumulation by collapsing the proton motive force that energizes the efflux mechanism. A comparison with results obtained with the lysate experiment carried out on the bacterial population produced similar results demonstrating the robustness of this new method. Uptake and efflux are independent of external concentration of compound under the conditions used. However, efflux is dependent on the rate of internal accumulation of compound and it may be overcome if uptake of antibiotics is very efficient. Table S3 presents the effect of CCCP in the presence of several concentrations of compound **1** incubated with bacteria. Using a standard curve, we were able to estimate the concentration of compound **1** inside the cells and the number of molecules taken up by a single bacterium. As hypothesized, with higher external concentrations of fleroxacin we observed a saturation of the efflux activity (compare the ratio with/without CCCP).

In contrast to the lysate protocol that studied bacterial populations, the bacterial suspension that was used to study individual bacteria could not be washed to eliminate compound that was adsorbed on the outer membrane. This protocol difference might have led to the differing results generated by the two methods in the case of compound **3**. The lack of removal of compound adsorbed to the cell surface, the lack of a suitable internal control, and the difficulty in measuring a precise time 0, are all aspects that we will look to solve in the future by developing a combined microfluidic device assay. In theory, using this method we should be able to measure (i) the drug influx and efflux by rinsing with different drugs, and (ii) the kinetic constant for each bacterial transporter involved in drug translocation.

Modeling of the bacterial membrane has brought about new information and metrics concerning the diffusion of molecules through the lipid bilayer or through single porins, and is important to complement our understanding of the uptake measured for each bacterium in the current study^8^,^34^,^35^. The ultimate goal is the ability to quantify drugs inside the bacteria and to determine the residence time with the antibiotic target. The method of antibiotic quantification inside isolated bacterial cells, as described by the study, is now ready for application to antibacterial molecules that fluoresce after DUV excitation. This method is applicable beyond antibiotics for any small molecules with the appropriate spectroscopic properties. Development of new methodologies for time lapse and 3D imaging to follow the intracellular localization of antibiotics are also planned for the future.

Using this new method we are now able to discriminate the key steps that govern the threshold of intracellular accumulation required for antibacterial activity and show the involvement of efflux in this process. As expected, when we compared the slope and the accumulation rate for compounds **1** and **3** in EA298 versus EA289, the intracellular concentration of the two molecules were more higher in the TolC- strain compared to the efflux overproducer. Moreover, we observed a faster accumulation of compound **1** in EA298 than that for compound **3**, suggesting a difference in drug influx (compounds 1 and 3 are both of the fluoroquinolone class of antibiotics). This method opens the way to follow the accumulation of different compounds in bacterial cells expressing various phenotypes such as porin alteration, efflux production, etc, and consequently to define the respective contribution of different pharmacophoric groups involved in the transport through bacterial membranes including influx and efflux.

It is important to mention that this method can be apply to other classes of antibiotics in addition to ciprofloxacin, norfloxacin and other fluoroquinolones. Recently described fluorescent derivatives of ß-lactams^36^,^37^ can be assayed by using this method to follow the uptake and the accumulation inside individual susceptible and resistant bacterial cells. Moreover, we may also develop a competitive approach in which the extent of accumulation of fleroxacin or other fluorescent antibiotic will be measured in the presence of various concentrations of the tested non-fluorescent molecule. This will allow the method to be include to non-fluorescent compounds, further extending the SIAR concept.

On the basis of our findings, we suggest that translocation through the bacterial membrane (influx) and the activity of efflux pumps (efflux), should be jointly addressed to fully understand the required intracellular concentration of antibiotic close to the target. The implementation of this method should focus on pharmacophoric groups involved in bacterial membrane translocation and generate Structure-Intracellular Accumulation Relationships (SIAR) as a new concept for future study regarding the drug translocation through biological membranes. This would enable the rational design of new antibiotic molecules containing the appropriate modifications to promote efficient intracellular accumulation of antibacterial molecules. For the first time, it becomes possible to visualize the accumulation rate of an antibacterial agent, to determine its concentration inside the bacterial cell and to potentially correlate this data with the measurement of the bacterial susceptibility in a same sample. In addition this method could be further improved to focus on the sub-cellular localization of the antibiotic molecules inside various bacterial compartments such as periplasm or cytoplasm.

## Methods

### Antibacterial activity

The minimal inhibitory concentrations (MICs) were determined by broth dilution method as previously described^21^. Susceptibilities were determined in 96-well microplates with an inoculum of 2 × 10^5^ cfu in 200 μL of Mueller Hinton II broth containing two-fold serial dilutions of each antibiotics or compounds. MICs were determined in the absence and in the presence of a membrane permeabilizer, the polymyxin B nonapeptide (PMBN) used at 51.2 mg/L (1/5 or 1/10 of its direct MIC previously determined) or an efflux pump substrate, the phenylalanine arginine β-naphthylamide (PAβN) used at 20 mg/L. Compound **1** was used as internal antibiotic reference. The MIC was defined as the lowest concentration of each compound for which no visible growth was observed after 18 h of incubation at 37 °C. Each test was performed in duplicate or triplicate. Results were expressed in mg/L.

### Compound accumulation

Two *Enterobacter aerogenes* (*E. aerogenes*) strains previously described were used: EA289 was a Kan^s^ derivative of an *E. aerogenes* multi-drug resistant (MDR) clinical isolate overexpressing AcrAB efflux pumps, and EA298 its *tol*C^-^ derivative^20^.

Bacteria grown at 37 °C in Luria-Bertani broth in its exponential-phase (corresponding to 0.6 optical density units at 600 nm) were concentrated 10-fold. Briefly, the bacterial suspension was centrifuged at 6 000 g for 15 min at 20 °C and pellets were re-suspended in 1/10 of the initial volume in a sodium phosphate buffer (50 mM) at pH 7 supplemented with MgCl_2_ (5 mM) (NaPi-MgCl_2_ buffer) to obtain a density of 10^10^ CFU.ml^−1^. 1.6 ml of the bacterial suspension was incubated 30 min at 37 °C (final volume 2 ml) with each compound: **1** at 64 mg/L, **2** at 2 mg/L and **3** at 16 mg/L, in the absence or in the presence of the efflux blocker carbonyl cyanide m-chlorophenylhydrazone (CCCP), used at 10 μM that collapses the energy-driven force needed by the efflux pump^21^. Bacterial suspensions incubated without antibiotics, with or without CCCP, were used as controls. Suspensions (800 μl or 400 μl) were then loaded on 1 M sucrose cushions (1100 μl or 550 μl respectively) and centrifuged at 13 000 rpm for 5 min at 4 °C to eliminate extracellular-adsorbed compounds and collect washed bacteria. To control that the bacterial cells are alive during the experimental time, we determined the number of colony-forming-unit (CFU) by sampling the bacterial suspension at 5, 10 and 15 min during antibiotic incubation. CFUs were determined along the experiment and no change in cell viability was observed during this period that corresponds to accumulation assay (data not shown). It must be noted that the ratio cell/antibiotic concentration were different in the MIC assay and in accumulation assay carried out using starving conditions during a limited incubation time (15–20 min).

DNA gyrase is the primary target of quinolones in Gram-negative organisms such as *E. aerogenes* and so it is important to mention that the strains used in this study contain a mutation in the DNA gyrase that renders them resistant to compounds **1** and **3**^16^,^20^,^21^.

### Bacterial population accumulation

To follow the compound uptake by bacterial population, we used the routine fluorimetric method previously described by Chapman *et al.*^15^. Briefly, pellets corresponding to 800 μl of bacterial suspensions were lysed with 500 μl of 0.1 M Glycin-HCl pH3 buffer for 2 h at room temperature. After a centrifugation for 15 min at 13 000 rpm at 4 °C, 400 μl of lysates were diluted in 600 μl of 0.1 M Glycin-HCl pH3 buffer and analysed by spectrofluorimetry.

### Individual bacterium accumulation

To detect the antibiotics’ fluorescence from single bacteria, pellets corresponding to 400 μl of bacterial suspensions used for the simultaneous accumulation assays (see above, section Compound accumulation) were re-suspended in 100 μl of NaPi-MgCl_2_ buffer. 0.5 μl of resuspended pellets were deposited between two quartz coverslips and analysed by DUV microspectrofluorimetry or DUV fluorescence imaging.

UV is known to kill bacteria upon prolonged exposure. We evaluated the cell viability under the same irradiation conditions by using CFU determination.

We tested EA289 + compound **1** versus EA289 + compound **1** + UV irradiation with the following times of incubation: 5, 10, 15 min. Small volumes were collected and dilutions were performed and plated on petri dishes. CFUs were measured and survival ratios, with and without irradiation, were generated. The assays were performed in triplicate. No decrease of cell survival was observed (Supplementary Table S2). Moreover, it must be noted that only microwatts of UV are deposited onto the sample, a dose way lower than the UV dose used for cells killing.

### Time course of individual bacteria accumulation

Bacterial cultures were grown in LB broth until exponential phase (OD = 0.6), centrifuged 15 min at 20 °C at 6 000 g and re-suspended with NaPi-MgCl_2_ buffer to obtained an OD = 0.48. Then 120 μl were centrifuged 5 min at 4 °C at 13 000 rpm on a 165 μl of 1 M sucrose cushion. Just before DUV microfluorimetry imaging analysis, the pellet was re-suspended with 30 μl of NaPi-MgCl_2_, with or without CCCP at 10 μM, and/or compounds: **1** at 128 mg/L, **2** at 8 mg/L or **3** at 64 mg/L, and 0.5 μl were deposited between two quartz coverslips. Contrary to individual bacterium accumulation where centrifugation on sucrose cushion can be done to eliminate extracellular-adsorbed compounds, we have currently no way to wash the extracellular content. Background intensity should raise but the concentration we are using is not high enough to be detected.

### Spectrofluorimetry Analysis

The fluorescence spectra of bacteria lysates were recorded at 20 °C using a FluroMax-4 (HORIBA Jobin Yvon INC, Chilly Mazarin, France) spectrofluorimeter. A 0.5 cm pathlength quartz cuvette was used for measurement. Fluorescence emission spectra of lysates were recorded at an excitation wavelength of 290 nm for compound **1**, or 275 nm for compounds **2** and **3**. These wavelengths were previously determined to be suitable for compounds detection (*e.g.* in 0.1 M buffer of Glycin-HCl of pH = 3)^16^.

To quantify the compound fluorescence intensity in bacteria lysates, spectra were normalized using the tryptophan peak at 356 nm before subtraction of control spectra (no compound, with or without CCCP only). Compound concentrations in bacteria lysate were calculated according to a calibration curve generated by mixing a known concentration of each compound with bacterial lysate in Glycin-HCl at pH = 3.

### DUV Microspectrofluorimetry

Fluorescence spectra from individual bacteria were recorded on a deep ultraviolet (DUV) microspectrofluorimeter at Synchrotron SOLEIL^17^. Briefly, the emission of the bending magnet from DISCO beamline^38^ was monochromatized at 275 or 290 nm before focalization on bacteria deposited with their medium (0.5 μl of resuspended pellets) on quartz coverslips). The objective used was a Zeiss ultrafluar glycerin immersion objective with 100× magnification. Emission spectra were recorded during 60 s at an excitation wavelength of 290 nm for compound **1**, or 275 nm for compounds **2** and **3** and have been measured on 5 different localizations (*e.g.* 5 different bacteria). Individual spectra recorded in the same condition from each bacterium were assembled, baseline corrected, and normalized on the tryptophan peak at 340 nm before averaging.

### DUV Fluorescence Imaging

Bacterial strains were observed in brightfield and excited in DUV with a Zeiss Axio-observer Z1. The selected objective was a 100× Zeiss ultrafluar objective needing glycerine immersion.

Fluorescence was recorded following excitation at 290 nm for compound **1**, or 275 nm for compounds **2** and **3**, reflected by a 300 nm dichroic mirror (OMEGA Optical, Inc., USA) before selection by emission bandpass filter at 323–353 nm for the bacterial autoflorescence and 370–410 nm for compound **2** or 420–480 nm wavelengths for compounds **1** and **3** (OMEGA Optical, Inc., USA). Fluorescence images were recorded with a PRINCETON PIXIS 1024-BUV EM-CCD camera (Princeton, USA). In order to widen the dynamic range, the integration time of the camera was 30 sec for the fixed excitation and filter combination used to visualize the compounds **3** and **2** fluorescence. For each condition, data were collected from three to four different localizations (about 100 bacteria total) were acquired. The imaging system was controlled under μManager^39^. The dose of DUV irradiating the sample was in the low μW range and no increase in cell death was observed. To control that the bacterial cells are alive during the experimental time in the presence of fleroxacin, we determined the number of CFUs as previously described (see above).

The image analyses were performed with Image J (Rasband, W.S., ImageJ, U. S. National Institutes of Health, Bethesda, Maryland, USA, http://imagej.nih.gov/ij/, 1997–2011). A specific plug-in has been written to standardize the analysis.

## Additional Information

**How to cite this article**: Cinquin, B. *et al.* Microspectrometric insights on the uptake of antibiotics at the single bacterial cell level. *Sci. Rep.* **5**, 17968; doi: 10.1038/srep17968 (2015).

## Supplementary Material

Supplementary Information

## Figures and Tables

**Table 1:** Antibacterial activities and spectral properties of compounds.

C	**MIC**								**Spectral properties**	
	**EA289 (*****acr*****AB+)**				**EA298 (*****tol*****C–)**				**Wavelength (nm)**	
	–	+PMBN	+PAßN	+CCCP	–	+PMBN	+PAßN	+CCCP	excitation	emission
C1	64	64	32	64	8	8	8	8	290	460
C2	8–4	1–0.5	0.25	4	0.125	0.06	0.06	0.06	247, 292, 360	415
C3	>128	128	64–32	128	8	8	16	8	275, 320	435

MIC (Minimal inhibitory concentration) are indicated in mg/L; PMBN (Polymyxin B nonapeptide) was used at 51.2 mg/L, PAβN (Phenylalanine-arginine-β-naphthylamide) at 20 mg/L and CCCP (carbonyl cyanide *m*-chlorophenylhydrazone) at 10 μM. C1, compound **1**; C2, coumpound **2**; C3, compound **3**.

**Table 2:** Comparison between lysate and kinetic assays.

		**Lysate assay**	**Kinetic assay**
C1	EA289	100%	100%
	EA298	191%	175%
C3	EA289	100%	100%
	EA298	302%	430%
C2	EA289	100%	100%
	EA298	385%	110%

% of the accumulation obtained by lysate or kinetic assays of the compounds **1**, **2** or **3** in EA298 strain devoid of TolC after normalization by accumulation in EA289 efflux-overproducer. C1 – C3 correspond to compound **1** to compound **3** respectively.
