# Supplementary material for: Microspectrometric insights on the uptake of antibiotics at the single bacterial cell level
Source: Sci Rep. 2015 Dec 11;5:17968. doi: 10.1038/srep17968 (PMC4675965; doi:10.1038/srep17968)
Supplement: Supplementary Information [file srep17968-s1.doc]

**Supplementary tables and Figures**

**Microspectrometric insights on the uptake of antibiotics at the single bacterial cell level**

Bertrand Cinquin^1,5^, Laure Maigre^2,5^, Elizabeth Pinet^2^, Jacqueline Chevalier^2^, Robert A. Stavenger^3^, Scott Mills^4^, Matthieu Réfregiers^1,6^, Jean-Marie Pagès^2,6^

^1^, DISCO beamline, Synchrotron Soleil, Saint-Aubin, France

^2^, UMR-MD1, Aix-Marseille Université, IRBA, Marseille, France

^3^ Antibacterial DPU, GlaxoSmithKline, Collegeville PA, USA

^4^ Infection Bioscience, AstraZeneca R&D Boston, Waltham, Massachusetts, USA

^5^ these authors contributed equally to this work

^6^ These authors jointly directed this work

Table 1S: Comparison between lysate and kinetic assays

% of the accumulation obtained by lysate or kinetic assays of the compounds **1** or **3** in presence of CCCP in EA298 strain devoid of TolC after normalization by accumulation in EA289 efflux-overproducer.

|  |  | **Lysate assay** | | **Kinetic assay** | |
| --- | --- | --- | --- | --- | --- |
| **Compound 1 + CCCP** | **EA289** | 203% | 196% | |  |
|  | **EA298** | 213% | 212% | |  |
| **Compound 3 + CCCP** | **EA289** | 328% | 467% | |  |
|  | **EA298** | *Nd* | 467% | |  |

Nd: not determined

Table 2S: Control of survival cells during irradiation assays.

Survival percentage of EA289 bacterial cells incubated in the presence of fleroxacin 8 µg/ml was measured under discontinuous UV irradiation at selected times according to the protocol used for individual bacterial accumulation. The ratio of survival cells was calculated as % to the control performed under the same conditions without DUV irradiation. The total experimental and the irradiation times are indicated for each determination. Each test was performed in duplicate.

| **Total experiment time (min)** | 5 | 11 | 17 |
| --- | --- | --- | --- |
| **DUV exposure time (min)** | 3 | 6 | 9 |
| **Survival bacterial cell (% of the control)** | 98 | 100 | 100 |

Table 3S: Quantification of accumulated compound

Compound 1 was incubated at different concentration with EA289 during 30 min in the absence or presence of CCCP. Lysates were observed by DUV spectroscopy as described in the method section. A standard range was used to correlate with concentration. Optical density of bacteria being known, number of molecules accumulated by a single bacterium can be extrapolated. Data with * are coming from previous work^16^.

Figure S1: Compounds fluorescence emission spectrum. Fluorescence emission spectrum of a solution of compound **1** excited at 290 nm, compound **2** excited at 275 nm and compound **3** excited at 275 nm in a HCl Glycin solution at 0.75 µM. The spectrum has been subtracted by a solution of HCl Glycin.


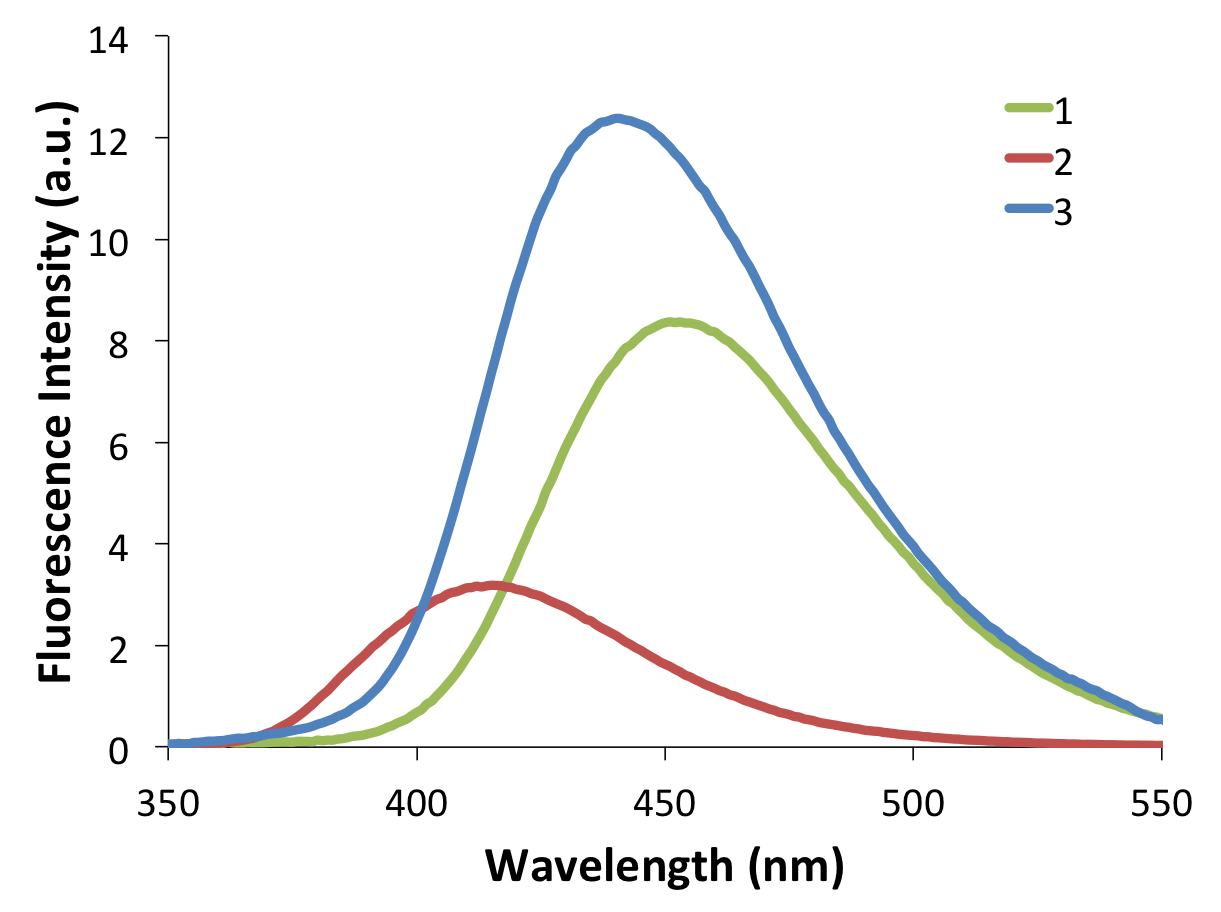


Figure S2: CCCP effect on compound fluorescence. Emission spectra of a solution of CCCP (10 µM), a solution of compound 1 at 8 µg/mL and a solution of compound 1at 8 µg/mL with CCCP (10 µM) excited at 290 nm (nominal excitation wavelength for compound 1). No effect of CCCP is noticeable.

Figure S3: Time course of bacterial accumulation. Average fluorescence intensity of 100 individual EA289 (plain curve) bacteria or EA298 (dashed curve) incubated with compound **1** (**a**), with compound **2** (**b**) or compound **3** (**c**) in the absence or in the presence of CCCP (dotted curve for EA289, dot-dashed curve for EA298).
